# Variations in contact patterns and dispatch guideline adherence between Norwegian emergency medical communication centres - a cross-sectional study

**DOI:** 10.1186/1757-7241-22-2

**Published:** 2014-01-08

**Authors:** Eirin N Ellensen, Steinar Hunskaar, Torben Wisborg, Erik Zakariassen

**Affiliations:** 1 Department of Research, Norwegian Air Ambulance Foundation, P.O. Box 94, N-1441, Drøbak, Norway; 2 Department of Global Public Health and Primary Care, University of Bergen, P.O. Box 7804, N-5020 Bergen, Norway; 3 National Centre for Emergency Primary Health Care, Uni Health, Uni Research, Kalfarv. 31, N-5018 Bergen, Norway; 4 Anaesthesia and Critical Care Research Group, Faculty of Health Sciences, University of Tromsø, N-9037 Tromsø, Norway; 5 Norwegian Trauma Competency Service, Oslo University Hospital, N-0450 Oslo, Norway

**Keywords:** Criteria based dispatch, Emergency medical dispatch, Emergency medical services, Emergency medical communication systems, Triage, Guideline adherence

## Abstract

**Background:**

The 19 Norwegian Emergency medical communication centres (EMCCs) use Norwegian Index for medical emergency assistance (Index) as dispatch guidelines. Little is known about the use of Index, nor its validity. We aimed to document the epidemiology of contacts made to the public emergency medical phone number and the operators’ self-reported use of Index as a first step towards a validation study.

**Methods:**

We registered all medical emergency calls to the EMCCs during a 72 h period in a national cross sectional study. We subsequently sent a questionnaire to all EMCC operators in Norway, asking how they use Index. A combined outcome variable “use of Index” was computed through a Likert scale, range 1–5. Regression models were used to examine factors influencing use.

**Results:**

2 298 contacts were included. National contact rate was 56/1 000 inhabitants per year, range between EMCCs 34 – 119. Acute contact (life-threatening situations) rate was 21/1 000 per year, range between EMCCs 5 – 31. Index criteria 6 – ’Unresolved problem’ accounts for 20% of the 113 contacts, range between EMCCs 10 – 42%. The mean use of Index was 3.95 (SD 0.39), corresponding to “more than 75% of emergency calls”. There were differences in use of Index on EMCC level, range 3.7 – 4.4, and a multi regression model explained 23.4% of the variation in use. Operators working rotation with ground ambulance services reported reduced use of Index compared to operators not working in rotation, while distinct EMCC focus on Index increased use of Index compared to EMCCs with no focus on Index. Use of electronic records and operators experience were the main reasons given for not using Index.

**Conclusions:**

There is a large variation between the EMCCs with regard to both contact patterns and use of Index. There is a relatively high overall self-reported use of Index by the operators, with variations on both individual and EMCC level.

## Introduction

Norway has a dedicated medical emergency phone number 113, which is answered and handled by an operator at the nearest Emergency medical communication centre (EMCC). The EMCCs dispatch the ambulance fleet, consisting of cars, boats, helicopters and planes, and facilitates radio and telephone communication amongst all the different participants in the chain of pre-hospital emergency medical care, from ambulance personnel through general practitioners on call to in-hospital specialists, and with other emergency authorities like police and fire brigades [1].

Norwegian Index for Medical Emergency Assistance (Index) [2] is the dispatch guidelines used by the EMCCs. It was developed from the Criteria Based Dispatch (CBD) guidelines in King County, Washington, USA [3], and introduced in Norway in 1994. Today Index is implemented as the only dispatch tool in all 19 EMCCs, serving an important role in securing equal high quality of the emergency health communication system throughout Norway [2,4,5], as well as assisting the individual operator when handling an emergency call. Despite its importance on both the individual level (the patient in acute need of medical assistance) and on the systemic level (adequate resource allocation), Index has not been evaluated or validated during its nearly twenty years in use.

The CBD guidelines, and thus the Index, use symptom criteria to determine the urgency of the medical condition, and the appropriate level of response. This allows for a dynamic approach to the dispatch situation, compared to the more strict protocol compliance required by algorithm based dispatch systems. CBD and Index hence require an operator with a certain level of medical education and experience. In Norway, this is normally a registered nurse with experience from an emergency ward or an intensive care unit. The interpretation and modification of the formal guidelines according to individual skills will influence the criteria set, introduce an unknown variance in guideline adherence and hence lower the validity of any study regarding dispatch [6]. Guideline adherence is defined as “conformity in fulfilling or following official, recognized, or institutional requirements, guidelines, recommendations, protocols, pathways, or other standards” [7]. The algorithm based dispatch systems, such as Medical Priority Dispatch System (MPDS), traditionally use the term ’compliance’ when referring to protocol adherence [6,8,9]. MPDS monitor compliance frequently along with many other quality markers [9], facilitating validity studies.

To our knowledge there are no international studies addressing CBD adherence. A recent systematic review on adherence to guidelines and protocols in the pre-hospital setting was not able to identify any eligible study in the emergency medical dispatch setting, neither CBD nor MPDS based [10]. However, a Norwegian study from 2005 evaluated dispatch in drug-related emergencies, and found an Index adherence of 99% based on multiple choice questionnaire, but only 64% guideline adherence based on log recordings [11].

A study on the epidemiology of medical emergency contacts made to three Norwegian EMCCs during a three month period in 2007 [12], focused on acute life threatening situations coded as acute only. The study showed statistically significant differences in the acute response rates among the EMCCs, but gave no explanation with regards to whether these differences could indicate variations in EMCC triaging or differences in the populations. National data on different urgency levels, use of Index criteria codes and contact rates to explore possible variations on a national level or study the triaging component further was not available in 2011 when we started the present study, but is now available as an annual report [13].

Before starting a validation study we needed to document the epidemiology of the contacts made to 113 and the emergency medical dispatch guideline adherence. The first aim of this study was to document possible differences in urgency levels, Index criteria and contact rates among the EMCCs. The second aim was to document the operators’ self-reported use of Index by a questionnaire, and determine factors influencing the use.

## Methods and materials

### Setting

Autumn 2011 there were 19 EMCCs in Norway, belonging to four regional health trusts. The EMCCs differ in size, covering from 65 000 to 1 165 000 inhabitants, with nearly 5 million inhabitants in total [14,15]. They are traditionally manned by registered nurse operators who answer and handle the 113 calls, and ambulance educated resource coordinators who dispatch and coordinate the ambulance fleet.

In addition to using the same dispatch guideline to prioritize and handle contacts [2], all EMCCs use the same software program Acute medical information system (AMIS) to register information and document each contact. AMIS contains information on Index criteria used, urgency level, and response dispatched.

### 113 Epidemiology – cross sectional population based survey

**Figure 1:**
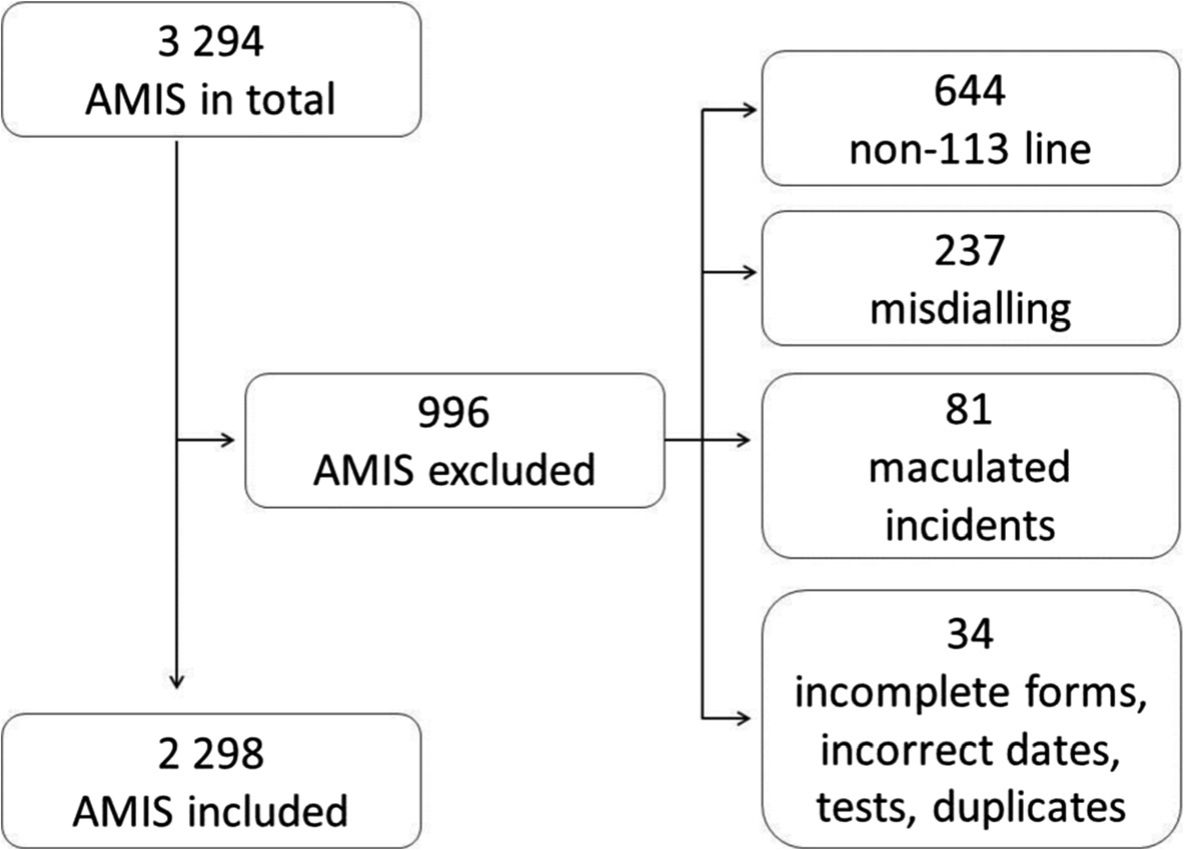
Excluded AMIS forms.

All 19 EMCCs contributed with data from every medical emergency contact made to the emergency medical phone number 113, during a 72 hour set period in August 2011. The data were collected in form of AMIS printouts, and contained information about date, time, the caller role, the patients sex and age, Index criteria used, urgency, response, and involvement of ambulances, air ambulances, GPs and others for each contact. Only calls registered to the 113 telephone line was included, excluding calls made by direct lines from fire brigades, police and Local emergency communication centers (LEMCs). Duplicates, maculated incidents and misdialing were also excluded (Figure 1).

### Norwegian index for medical emergency assistance (index)

Index is a flip-over in large format consisting of a Start page, 36 symptom cards and 4 administrative cards. The Start page is built like an algorithm and clarifies important information as location, phone number, vital functions and a brief questioning on the patient’s problem. The operator then moves on to the proper symptom card, determined by the problem presented. The symptom cards display a list of different criteria in decreasing severity, from the acute symptoms at the top and all the way down to the non-urgent symptoms at the bottom. The operator starts at the top and moves downwards through specific criteria, until one criterion is finally met. This criterion then determines the response dispatched. Index divides the response into three categories; red, yellow and green. Red response is when the situation is acute and life threatening, and calls for immediate “blue lights and sirens” ambulance dispatch and GP on-call alarm. Yellow response is an urgent and potentially life-threatening situation where ambulance and GP alarm is constantly assessed. Green responses are non-urgent situations, where the patients often are referred to the primary level of emergency care, unless there is an obvious need of ambulance transport.

### Self-reported use of index – questionnaire based survey

There were no validated questionnaires available to address our aim, so we developed a questionnaire in cooperation with one of the EMCCs and tested it on operators there to avoid possible misunderstandings.

Questions on their use of Index were arranged as a Likert scale of five symmetric response formats; never, seldom (<25%), sometimes, often (>75%) and always. The eleven different questions were all expressed in the same form: “During a real emergency call, how often do you…?” and covered different aspects of Index (Table 1). In addition to asking directly about use of Start page, we also asked specific questions from the Start page, like “… clarify if the patient is awake?” and “… if the patient can talk?” to unmask actual use of Start page and not just flipping of the page. The operators were asked about their educational level, EMCC work experience, initial training and later repetitions with regards to use of Index. We also asked if they thought there was focus on use of Index at their EMCC. The operators were given the possibility to add explanations or points of view at the end of the questionnaire. The questionnaire was distributed in paper through EMCC management, but returned directly to the research facility to secure anonymity for the individual operator.

**Table 1:** Questions used to determine use of Index

	**N**	**Mean**	**SD**
**When receiving a true emergency medical call, please specify how often you do the following:**			
I use the table format Index during the conversation	270	4.02	0.82
I use Start page	272	3.36	1.23
I clarify if the patient is awake	270	4.93	0.26
I clarify whether the patient can talk or not	270	4.32	0.73
I do not decide upon urgency before criteria (reversed in questionnaire)	272	3.02	0.87
I do not determine criteria code without using Index (reversed in questionnaire)	269	3.80	0.94
I start on top of the symptom card and work my way downwards	267	4.01	0.86
I ask further questions	269	4.09	0.74
I give advices to the caller/patient, if needed	270	4.56	0.56
I give advices to health personnel on scene, if needed	268	3.70	1.00
If Index is not used during the call, I check afterwards whether I have covered everything	263	3.63	1.02
Total (Likert score)		3.95	0.39

1 = Never, 2 = Seldom (<25%), 3 = Sometimes, 4 = Often (>75%), 5 = Always. N = 272 operators.

### Population

There were 429 persons working as operators in the EMCCs when our study started. An operator was defined as the person for whom the primary occupation is to answer the emergency medical phone call. A resource coordinator was defined as a person for whom the primary occupation is to coordinate the ambulance fleet. Only questionnaires from operators were included in the study.

### Statistical analysis and ethical approvals

Continuous data are presented as mean (SD) or median (quartiles) for symmetric and skewed data, respectively. Rates are calculated as contacts per 1 000 inhabitants per year. The five response formats on the Likert scale were valued 1 to 5, and the outcome variable “use of Index” was calculated for each operator as the mean score from all eleven questions constituting the Likert scale. Q-Q plots were used to check the outcome variable for normal distribution. We explored the variation in mean use of Index with respect to each of the explanatory variables by one-way analysis of variance (ANOVA) and univariate linear regression. All explanatory variables were then entered in a full multiple linear regression model. Effect estimates are reported with 95% confidence intervals (CI). A possible hierarchical effect of EMCC central was assessed by adjusting for central in a mixed model. There were only marginal differences in effect estimates when adjusting for the mixed effect, and we therefore consequently report results from the ANOVA and linear regression models.

The statistical analyses were performed using Statistical Package for the Social Sciences (IBM SPSS Statistics 20) and STATA (Stata/IC 12.1). P-values below 0.05 were considered statistically significant.

Both studies were evaluated by the Regional committees for medical and health research ethics and considered not to be in need of ethical assessment. Exemption for consent was given by the Regional committees for medical and health research ethics in terms of receiving and analyzing sensitive data. The Data Protection Official for Research approved the study.

## Results

### Epidemiology

We collected 3 294 AMIS forms during a 72 hour period, of which 2 298 were included for analyses. This gives a calculated overall national emergency medical contact rate of 56/1 000 inhabitants per year (95% CI 54–59), ranging from 34 (95% CI 22–46) to 119 (95% CI 91–146) among the different EMCCs. Of the 2 298 contacts 37% were assessed to be acute, 34% urgent, and 27% non-urgent, while 1.8% of the contacts had no urgency assessment. Looking at acute contacts separately, the rates varied significantly from 5 to 31/1 000 inhabitants per year (Table 2).

**Table 2:** Acute contact rates between different Emergency medical communication centres

**EMCC**	**Acute contacts**	**Acute contacts**	**95% CI**
	**N**	**Rate**	**Rate**
1	19	31	(17–46)
2	14	14	(7–22)
3	11	21	(8–33)
4	13	12	(5–18)
5	3	5	(0–10)
6	17	15	(8–22)
7	46	19	(13–24)
8	12	13	(6–21)
9	18	15	(8–22)
10	12	14	(6–21)
11	87	25	(20–31)
12	23	16	(10–23)
13	34	12	(8–16)
14	56	24	(17–30)
15	78	26	(20–32)
16	38	15	(11–20)
17	242	25	(22–28)
18	76	24	(19–30)
19	42	18	(13–24)
**Total**	841	21	(19–22)

Contacts made to all the EMCCs, and assessed by the operators to be of the most urgent of three urgency categories. N = number of acute contacts during a data collection period of 72 h. Rates calculated per 1 000 inhabitants per year.

**Figure 2:**
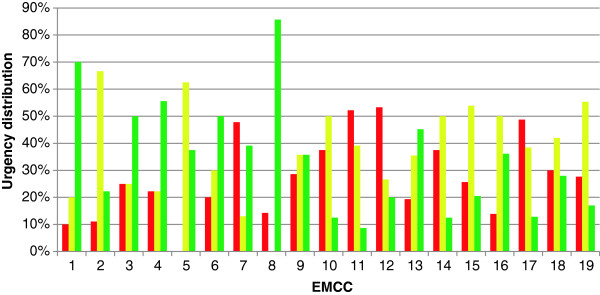
**Use of Index criteria “6 – Unresolved issue” between the EMCCs.** Differences in urgency assessment, and hence use of Index criteria «6 – Unresolved issue» between the different EMCCs. Red = acute, yellow = urgent, green = non-urgent contacts.

The most frequently used Index criteria was “6 - Unresolved issue” (Table 3), which accounted for 20% of all contacts. The use of this criteria differed from 10% at one EMCC to 42% at another. The use of other criteria like “5 – Ordered assignment” and “28 – Psychiatry – Suicide” also varied much between different EMCCs, from 0 to 22% and 19% respectively (Table 3). The urgency assessments on criteria 6 differed between the EMCCs; acute 0 – 53%, urgent 0 – 67% and non-urgent 9 – 86% (Figure 2).

**Table 3:** Top ten Index criteria

		Criteria number*												
**EMCC**	**Contacts**	**6**	**10**	**5**	**30**	**33**	**29**	**35**	**25**	**28**	**27**	**All other**	**Missing**	**Total**
	**N**	**%**	**%**	**%**	**%**	**%**	**%**	**%**	**%**	**%**	**%**	**%**	**%**	**%**
1	72	14	3	3	6	4	6	4	4	12	9	13	25	100
2	44	20	9	5	0	9	7	5	5	5	5	20	11	100
3	40	22	8	8	0	14	0	11	8	8	3	8	18	100
4	56	32	11	4	11	11	5	5	2	2	2	14	2	100
5	32	27	13	0	3	7	7	0	0	13	3	19	13	100
6	53	19	6	6	6	2	6	4	4	19	4	19	8	100
7	89	26	6	7	2	4	6	11	11	0	4	22	0	100
8	46	15	7	11	4	2	9	4	9	15	9	9	7	100
9	67	22	5	3	2	2	0	5	5	10	3	15	33	100
10	30	30	10	10	10	7	3	0	3	0	10	17	0	100
11	240	10	10	4	10	5	4	4	3	1	4	15	30	100
12	65	25	2	6	11	10	3	5	5	10	0	25	2	100
13	117	30	13	2	11	7	10	4	4	4	6	12	1	100
14	114	14	7	8	4	4	4	4	7	5	5	20	18	100
15	168	23	14	8	5	5	7	7	7	4	7	13	1	100
16	119	31	8	10	5	5	3	6	4	6	4	18	0	100
17	605	14	10	4	6	6	7	5	5	3	5	18	19	100
18	228	23	10	22	4	4	4	6	4	5	0	18	0	100
19	113	42	10	4	3	7	5	3	6	6	3	12	0	100
Total	2298	20	9	7	6	6	5	5	5	5	5	16	12	100

The most frequently used criteria in Index (%), for each of the 19 EMCC. All degrees of urgency included. N = total number of contacts during the study period of 72 h.

*Criteria number explanation: 5 – Commissioned assignments (by health personnel), 6 – Unresolved issue, 10 – Chest pain, heart disease, 25 – Stomach pain, back pain, 27 – Reduced consciousness, paralyses, 28 – Psychiatry, suicide, 29 – Breathing issues, 30 – Intoxication, overdose, 33 – Wounds, fractures, minor injuries, 35 – Accidents.

### Use of Index

We received 275 questionnaires after three reminders, of which 272 questionnaires were included in the study, representing all 19 EMCCs (response rate 63.4%). The operators were mainly female, registered nurses, of whom many worked in rotation at either emergency rooms (ER) or ambulances (Table 4). The majority received training in use of Index when starting to work at the EMCC, but later repetitions were rare (Table 4). The EMCC work experience ranged from 1 month to 36 years, with a median (quartile) time of 6 (3, 12) years.

**Table 4:** Operator characteristics

		**N**	**%**
Sex	Female	178	65
	Male	85	32
	Missing	9	3
		272	100
Education	Ambulance worker	50	18
	Registered nurse	230	85
	Ambulance worker and nurse	16	69
	Other	11	4
	Further education	86	32
Work rotation	None	73	27
	With emergency room	116	43
	With ambulance	44	16
	Other	19	7
	Missing	20	7
		272	100
Work experience	≤ 1 year	27	10
	>1 ≤ 5 years	95	35
	>5 ≤ 10 years	60	22
	> 10 years	78	29
	Missing	12	4
		272	100
Training in use of Index	Formal training from the start	234	86
	Formal training eventually	16	6
	Taught by colleagues	8	3
	None	2	1
	Missing	12	4
		272	100
Repetition of use of Index	Regularly	51	19
	Irregularly	129	47
	None	71	26
	Missing	21	8
		272	100

Background information on the operators’ (N = 272) educational and work experience, together with experienced initial training in use of Index and later repetitions.

**Figure 3:**
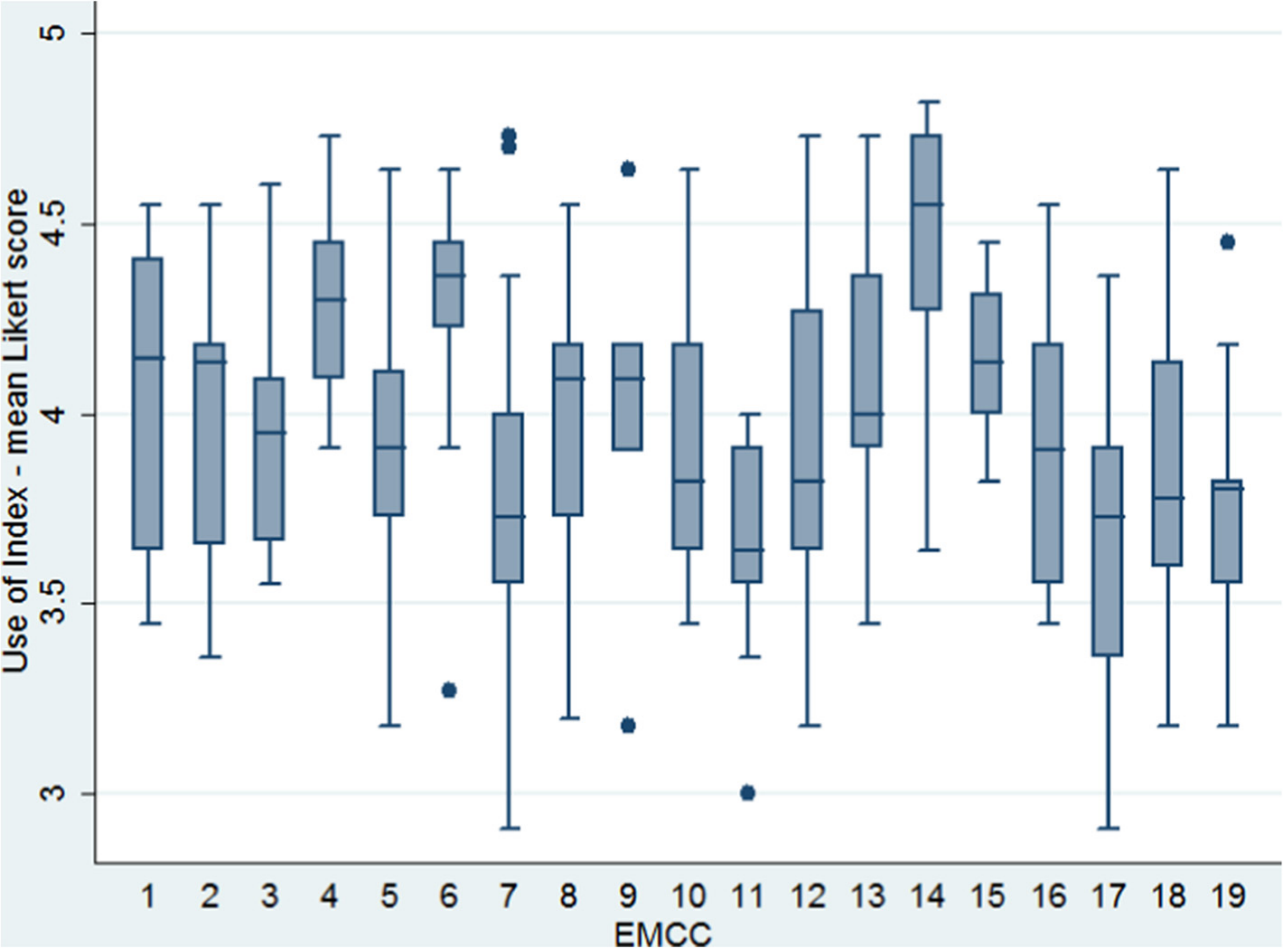
**Use of Index between the EMCCs.** Box plot of mean Likert-score values, displaying use of Index between the different EMCCs. The box represent the first, second (median) and third quartile of the data. The whiskers show the lowest datum within 1.5 interquartile range of the first quartile, and the highest datum within 1.5 interquartile range of the third quartile. Outliers are shown as dots.

Use of Index ranged from 2.91 to 4.82, with overall mean (SD) for all operators being 3.95 (0.39), corresponding closely to the response format “often, > 75%” (=4) in the questionnaire. The individual questions on use of Index ranged from 4.93 (0.26) for “I clarify if the patient is awake” to 3.02 (0.87) for “I do not decide upon urgency before criteria” (Table 1). ANOVA showed significant differences in use of Index among the EMCCs (p < 0.001), with the EMCCs ranging from 3.7 (0.24) to 4.4 (0.39). Figure 3 shows the spread between the EMCCs. Possible explanatory variables for use of Index were analyzed by univariate and multiple linear regression models (Table 5). Time, the effect of rotation with ambulance and a clear focus on use of Index at workplace on use of Index were statistically significant, with focus being by far the strongest component (β = 0.46, p < 0.001) (Table 5).

**Table 5:** Explanatory variables for the variation in use of Index

	**Univariate linear regression**			**Multiple linear regression**		
	**Estimate**	**(95% CI)**	**P-value**	**Estimate**	**(95% CI)**	**P-value**
Working experience	-0.011	(-0.018, -0.003)	0.004	-0.012	(-0.019, -0.004)	0.002
Rotation						
With ground ambulance	-0.218	(-0.360, -0.076)	0.003	-0.213	(-0.348, -0.078)	0.002
With emergency room	0.054	(-0.057, 0.165)	0.337	0.033	(-0.074, 0.139)	0.545
With others	-0.093	(-0.283, 0.098)	0.339	-0.046	(-0.229, 0.137)	0.621
Repetition of use of Index						
Regularly	0.214	(0.074, 0.354)	0.003	0.062	(-0.080, 0.205)	0.390
Irregularly	0.090	(-0.023, 0.203)	0.118	-0.002	(-0.116, 0.111)	0.969
Focus on use of Index						
Clear focus	0.522	(0.312, 0.732)	<0.001	0.459	(0.240, 0.679)	<0.001
Some focus	0.293	(0.073, 0.512)	0.009	0.207	(-0.013, 0.426)	0.065

Exploration of possible explanations for the variation in use of Index, by univariate and multiple linear regression models.

Working experience = effect per year. P-value < 0.05 was regarded as statistically significant. Explained variation, R^2^ = 0.234 for the multiple linear regression model.

For use of Start page separately, we found that 47% reported using the Start page often or always (> 75% of real emergency calls), while 93% always checked if the patient was awake and 46% if he could talk. The reasons given for not using Index are shown in Table 6.

**Table 6:** Reasons for not using Index or Start page

	**N**	**%**
**Question: When not using the Index flip over, why?**		
I find the criteria in AMIS	149	55
I know it	91	33
It takes too long	55	20
It is cumbersome to move my hands between Index and the keyboard	44	16
It has become a habit	24	9
I am expected to know it	23	8
It is of little help	13	5
I am not trained to use it	0	0
**Question: When not using the Start page, why?**		
I know it	117	43
I prefer to go straight to proper card	108	40
I find what I need in AMIS	57	21
It is cumbersome to move the hands between flip over and keyboard	38	14
It takes too long	27	10

Given reasons for not using Index/Start page, checked off by the operators. Total number of operators = 272.

The question “work percentage at EMCC” was misinterpreted by many operators with regard to whether include rotatory work at ER/ambulances or not, and was hence excluded from analysis. Explanations offered in free text did not add any relevant information on the questions addressed, and were not analyzed.

## Discussion

The main finding of the epidemiological part of the study was the wide spread between the different EMCCs, regarding both overall contact rates, specific acute rates and use of different Index criteria when assessing the situations. The questionnaire study showed a relatively high overall use of Index reported by the operators. It also showed large unexplained variations in the use of Start page and several symptom cards.

### Strengths and weaknesses of the studies

The epidemiology study brings new knowledge about contacts made to 113, while the self-reported use of Index represents the first step towards determining use of Index. Both studies include all 19 EMCCs and hence are representative on a national level.

The short study period of 72 h limits the validity of extrapolation, and was mainly due to capacity issues in both ends, as data had to be exchanged in paper format at the time being. The questionnaire was not validated, as we wanted the main study population as large as possible, in terms of both individuals and centers.

Self-reporting allows for over- and underestimation. This was a calculated risk as we aimed to document how the operators themselves thought they used Index, and the results must be interpreted as subjective. We will address the problem with subjective contra objective use of Index in future audio-log study. The response rate of 63.4% could represent a selection bias, but the large range in ’use of Index’ score, both on an individual level and on EMCC level, together with all EMCCs being represented, indicates that we have a representative material. The response rate can to some extent be explained by all information, distribution and reminders depending on the management at each EMCC. The official policy by the EMCC managements on always using Index, combined with the questionnaires and reminders being provided by the management, raises the possibilities of an “eagerness to please” bias. This was attempted opposed by ensuring anonymity and allowing the filled out questionnaires to return directly to the main investigator, through prepaid preaddressed envelopes following each single questionnaire. Recall biases are likely to occur when trying to recall what one usually does in certain situations. The use of a non-validated questionnaire led to misinterpretation of some questions. Never and always are narrow categories, and the results on use of Index might have been more correct if the categories had been more evenly distributed.

### Epidemiology

There is a wide spread in annual contact rates among the EMCCs, from 34 to 119/1 000 inhabitants. We have no national data on acute disease distribution to compare with, but one would not expect a similar spread in distribution. Accidents are shown to disperse a geographical pattern, a study from 2013 comparing a rural county with an urban/rural county found a higher accident rate in urban areas, but a higher mortality rate per accident in the rural areas [16]. This dispersion of accidents does not account for the wide spread in contact rates, and this could indicate that the population uses 113 differently depending on location. A previous study of geographic variations in alerting, dispatch and response found that while severity of illness/trauma had no effect on the use of 113, the use was lower in rural areas compared with urban areas [17]. This could be due to local differences in organisation of LEMCs and casualty clinics, and cultural differences for when to access the different levels of emergency health services.

When separating contacts assessed to be acute, we also find a large spread in rates between the different EMCCs. The overall acute contact rate of 21 is slightly lower than reported by a previous study based on data from three EMCCs. They found a three month acute rate of 6.2/1 000 inhabitants in 2007, giving an annual rate of 25/1 000 [18]. As our study shows that the EMCCs differ with regards to both urgency distributions and contact rates, differences in inclusion of EMCCs will affect these outcomes.

A recently published study from Denmark studied Danish Index’ ability to triage patients according to severity [19], following the implementation of the CBD guidelines in Denmark. They found a national acute (emergency level A) rate of 17 ambulance dispatches/1 000 inhabitants per year, which is somewhat lower than our 21 acute contacts/1 000 inhabitants per year. There were also differences between their included areas: with 13, 17 and 21 acute ambulance dispatches/1 000 per year. One possible explanation could be the large differences in EMCC population size, with five centres in Denmark and 19 in Norway covering 5.5 and 5 million inhabitants respectively. This difference could influence use of Index, and hence incidence of acute criteria codes. Cultural differences in what emergency medical level to access and differences in pre-hospital emergency medicine organization could influence this.

On primary health care level, a study of 85 000 contacts from 2007 investigated the distribution of urgency levels in the Norwegian emergency primary healthcare services: acute 2.3%, urgent 21.1% and non-urgent 76.6% [20]. Compared to our findings (acute 37%, urgent 35%, non-urgent 26%), these differences show that the population as a whole know what level to address, depending on degree of medical emergency.

The most frequently used Index criteria, all urgencies included, was “6 - Unresolved issue”. This criterion covers a whole range of situations; from the unclear situations where the operator gets too little information to choose another criterion to well-defined situations where no other criteria match. The large differences between the EMCCs, both in terms of total use of this criterion and the variation in urgencies as it was used in, clearly indicates a variation in use of Index from center to center.

### Use of Index

The operators reported a relatively high overall use of Index, corresponding to “in over 75% of real emergency calls”. The variation among the operators was quite large though. Although we found some factors associated with positive and negative effects on use of Index, we were only able to explain 23.4% of this variation, indicating that there are factors influencing use of Index that we were not able to uncover. The main reason given for not using Index was AMIS, and the confidence that this software program provided the necessary key words to properly assess the acute medical emergency situation. The operators’ background was to great extent as expected. It was an experienced group of employees, with median working time at the EMCC of 6 years. The results indicated decent routines for training new operators in use of Index, but not for repetition.

Our result of approximately 75% use of Index lies between the divergent findings from the 2005 study, with 99% self-reported Index guideline adherence and 64% adherence based on log-recordings [11]. To our knowledge there are no other studies addressing adherence to criteria based guidelines. As algorithm based dispatch systems require a much stricter adherence to protocol they have systems developed to monitor and increase protocol compliance, among other quality markers [6,8,21-24]. Although compliance is highlighted as such an important feature of MPDS, less than 3% of the registered users are Accredited Centers of Excellence [25], which among other quality measurements include a minimum of 90–95% compliance with different parts of the protocol [8].

Given the purpose of CBD guidelines, to assist health care personnel in decision making rather than defining specific questions or actions to be taken [3], use of Index was not expected to reach full score. Looking at the different explanatory variables though, it was rather unexpected that time working at an EMCC had so little effect on use of Index. It was equally unexpected that the effect of what EMCC the operator works at was so negligible, as we held this to be the natural explanation for the variations in use of Index.

Working in rotation with ground ambulance is the variable strongest associated with decreased use of Index. This is not associated with educational level, as half the operators rotating with ambulance are registered nurses and there is no difference in use of Index between the different educational levels among those rotating with ambulance. Previous research has found that individual experience and professional autonomy affects guideline adherence [10]. Our study finds no effect on use of Index for operators rotating with ER. A Dutch study on protocol adherence among emergency and ambulance nurses found the opposite result; ambulance nurses are more likely to hold protocol over experience while emergency nurses are more likely to hold experience over protocol [26].

The low use of Start page is reflected in the reasons given for not using it. They know it, and find the key words they need in AMIS screen. The Start page (3^rd^ edition) includes the phrase: “Is the patient awake and can talk?” [2]. The low percentage reporting to actually ask about this latter, confirms that neither AMIS nor memory equals Start page, and one may speculate that the reason for including this question is not fully understood among those using the tool.

The main reason for not using Index appears to be that the operators choose to use the AMIS screen above Index, and a belief that AMIS provides the necessary keys to assist them in properly assessing the situation. This might be of some concern, since AMIS does not supply any support in neither decision making nor advices for the public or health personnel at scene, and hence cannot replace Index. Furthermore, discarding the guidelines in favor of memory and own experience is a potential hazard of losing vital information or getting sidetracked due to unstructured interrogation. This risk is strongly advocated by critics of criteria based dispatch [9,22].

Based on our findings we would recommend increased focus on use of Index at each EMCC. The systematic review on guideline and protocol adherence in the pre-hospital and emergency care setting [10] finds that tailored strategies towards identified barriers improve professional practice, as well as strategies aimed at influencing factors improve guideline adherence. Evidence based recommendations and a relationship between guideline adherence and clinical outcome are also mentioned as important motivational factors for guideline adherence.

In this study we estimated a mean value for use of Index among operators in Norway, based on self-report, which obviously represents a subjective view. Investigating the real practice based on objective data is therefore a natural next step in documenting use of Index.

## Conclusion

The EMCCs varies regarding both contact rates and use of Index. The operators report use of Index in > 75% of real emergency situations, but there are large individual differences. Clear focus on use of Index at the EMCC was the strongest predictor for increased use, and work rotation with ground ambulance services was associated with decreased use.

## Abbreviations

AMIS: Acute medical information system; ANOVA: Analysis of variance; CBD: Criteria based dispatch; CI: Confidence interval; EMCC: Emergency medical communication centre; ER: Emergency room; GP: General practitioners; LEMC: Local emergency medical communication centre; MPDS: Medical priority dispatch system; SD: Standard deviation; SPSS: Statistical Package for the Social Sciences.

## Competing interests

The authors declare no competing interests. The funding foundation had no participation in conducting the study or writing the manuscript.

## Authors’ contributions

All authors contributed in designing the studies. ENE collected and analysed the data, and drafted the manuscript. All authors contributed substantially in the interpretation of the findings and rewriting the manuscript. All authors read and approved this final version.
